# Cleaning of Ultrafiltration Membranes: Long-Term Treatment of Car Wash Wastewater as a Case Study

**DOI:** 10.3390/membranes14070159

**Published:** 2024-07-19

**Authors:** Wirginia Tomczak, Piotr Woźniak, Marek Gryta, Joanna Grzechulska-Damszel, Monika Daniluk

**Affiliations:** 1 Faculty of Chemical Technology and Engineering, Bydgoszcz University of Science and Technology, 3 Seminaryjna Street, 85-326 Bydgoszcz, Poland; monika.daniluk@pbs.edu.pl; 2 Faculty of Chemical Technology and Engineering, West Pomeranian University of Technology in Szczecin, 10 Pułaskiego Street, 70-322 Szczecin, Poland; piotr.wozniak@zut.edu.pl (P.W.); joanna.grzechulska@zut.edu.pl (J.G.-D.)

**Keywords:** biofilm, car station, fouling, membrane cleaning, treatment, ultrafiltration, wastewater

## Abstract

Car wash wastewaters (CWWs) contain various pollutants with different contents. Hence, selecting an appropriate process for their treatment is a great challenge. Undoubtedly, the ultrafiltration (UF) process is one of the most interesting and reliable choices. Therefore, the main aim of the current study was to investigate the performance of the UF membranes used for the long-term treatment of real CWWs. For this purpose, two polyethersulfone (PES) membranes with molecular weight cut-off (MWCO) values equal to 10 and 100 kDa were applied. As expected, a significant decrease in the permeate flux during the UF run was observed. However, it was immediately demonstrated that the systematic cleaning of membranes (every day) with Insect agent (pH = 11.5) prevented a further decline in the process’s performance. In addition, this study focused on the relative flux during the process run with breaks lasting a few days when the UF installation was filled with distilled water. The results of this research indicated that aqueous media favor microorganism adherence to the surface which leads to the formation of biofilms inside processing installations. As a consequence, many attempts have been made to restore the initial membrane performance. It has been found that the application of several chemical agents is required. More precisely, the use of an Insect solution, P3 Ultrasil 11 agent, and phosphoric acid increases the relative flux to a value of 0.8. Finally, it has been indicated that the membranes used in this work are resistant to the long-term exposure to bacteria and chemical agents. However, during the separation of CWWs for the membrane with an MWCO of 10 kDa, a lesser fouling influence and higher effectiveness of cleaning were obtained. Finally, the present study demonstrates a novel analysis and innovative implications towards applying the UF process for the CWW treatment.

## 1. Introduction

Car washes are technical facilities that have been offering easy, practical and time-saving vehicle washing since 1939 [1]. However, according to the literature data, 200 L of water is used to wash one car [2] and 400 L of water is required to wash a bus [3]. Within this scenario, this service leads to the generation of huge amounts of wastewaters that contain various pollutants. Among them are grease, detergents, oil/water emulsion, sand-dust, organic matter, salts, surfactants and heavy metals [4]. Worthy of note, as pointed by Saad et al. [5], wastewaters from manual car wash stations are characterized by higher concentrations of pollutants compared to those from automatic ones.

Recently, Elgaali and Akram [6] indicated that usually, car wash wastewaters (CWWs) after a short pretreatment in holding tanks are discharged into the city sewer systems. More so, stations located far away from municipal sewer systems discharge wastewaters into nearby water courses. This, in turn, results in different harmful impacts on the environment [7]. Consequently, the market of CWW treatment is one of the fastest growing sectors in the area of business and technological solutions [8]. Indeed, in international practices, it is a key challenge from a viewpoint of environmental protection.

**Figure 1:**
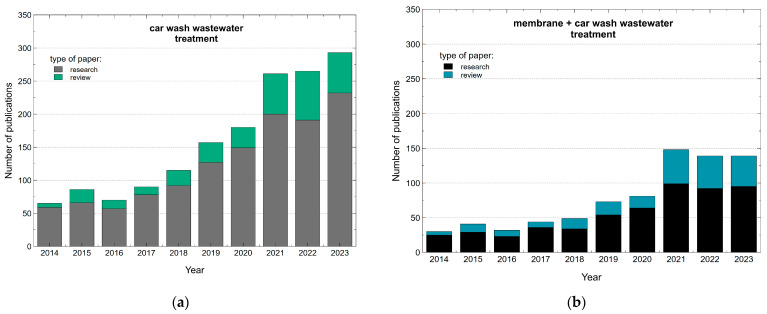
The number of publications according to Science Direct. Keywords: (**a**) car wash wastewater treatment; (**b**) membrane + car wash wastewater treatment. Data retrieved: 4 July 2024.

Correspondingly, substantial research has been carried out to determine both the physical–chemical parameters of CWWs and the effective processes of their treatment. The literature analysis has shown that the this field is currently highly studied. Moreover, in recent years, more and more scientific articles on the CWW treatment are published (Figure 1a). Indeed, the performed analysis demonstrated that over the last 10 years, 1251 research articles and 331 review articles devoted to this topic have been published (Science Direct search). This finding clearly indicates that 21% of the studies analyze the results of experimental work. Among these works, only about 40% have been related to membrane separation studies (Figure 1b). This limitation is clearly related to the fact that the compounds of CWWs lead to the membrane fouling, which is an increasingly important issue in the application of membrane processes. Furthermore, to the best of the authors’ knowledge, in the literature, there is no study presenting the possibilities of counteracting the fouling phenomenon. On the other hand, it should be noted that in the case of small manual washes, only simple and cheap membrane separators have a chance of being applied.

A significant difficulty in the separation of CWWs is the high variability of their composition. Examples of the CWW characteristics reported in the literature are presented in Table A1. It can be clearly seen that wastewaters are analyzed mainly in terms of pH, suspended solids (SSs), turbidity, chemical oxygen demand (COD), pH, concentration of total phosphorus (TP), grease and conductivity. It can be clearly stated that due to the presence of various pollutants with different contents, selecting an appropriate process for CWW treatment seems to be a great challenge [9].

Nowadays, different technologies and methods are proposed for the treatment of CWWs. They have been presented and discussed in detail in several review studies [10,11,12,13]. Among them are mainly (i) coagulation [14], (ii) electrocoagulation [15] and (iii) the membrane bioreactor (MBR) system [16] as well as integrated processes, for instance, (iv) sedimentation–coagulation [17] and (v) the coagulation–ozonation–MBR system [2]. However, the above-mentioned technologies are characterized by both high energy and high chemical consumption as well as waste production [18]. For these reasons, the above solutions are too complex and expensive to be applied in small hand car washes.

Compared to the conventional treatment technologies, a simpler solution can be obtained by using membrane processes. Indeed, Awad et al. [7], in their recently published review article, have pointed out that the application of ultrafiltration (UF) are the main focus points of membrane research in this sector. The literature review performed has indicated that the application of both polymeric [19,20,21,22,23,24,25,26,27] and ceramic [28,29] membranes for the separation of CWWs has been investigated. For instance, Istirokhatun et al. [26] have documented that the application of polyethersulfone (PES) membrane characterized by a molecular weight cut-off (MWCO) equal to 10 kDa ensures a complete rejection from CWW turbidity, as well as oil and grease. Moreover, in the above-mentioned study, the COD rejection was equal to 95%. In turn, in ref. [21], the CWWs were treated with the use of four UF membranes (1, 5, 10 and 50 kDa). It has been shown that the process decreased the initial COD from 314 ± 9.4 mg/L (Table A1) to values ranging from 64.5 ± 3.2 to 85.5 ± 4.3 mg/L depending on the membrane used. However, most published works mainly indicate the high efficiency of membrane separation and usually demonstrate experimental investigations performed for a few hours. Moreover, the most important impediment to implementing the UF process for CWW treatment is the flux reduction during the process run. Importantly, this issue is usually omitted in the presented works, although its solution determines UF application.

It is well known that the fouling phenomenon is caused by the blocking of membrane pores and/or deposition of cake layer on the membrane surface. It has been conclusively shown that the intensity of the fouling phenomenon depends on several factors, such as membrane pore size and structure, wastewater parameters including pH, concentration of oil and grease and petroleum materials [11]. In addition, CWWs contain various types of bacteria, including those resistant to antibiotics [30]. It is well accepted that microbiological pollutants present in CWWs may come from soil, mud and livestock waste [31]. Undoubtedly, microbiological risk assessment is a key factor when considering the reuse of the treated water from car wash stations [32]. It should be pointed out that complete microbiological analysis of wastewaters from car wash service stations have been widely reported in the literature [33,34,35]. As an example, in [33] *Bacillus subtilis*, *Morganella morganii*, *Aeromonas hydrophila* and *Citrobacter freundii* were detected in the CWW samples. In addition, in the above-mentioned study, it has been inferred that most of the bacteria species present in the analyzed samples can pose serious threats to both human health as well as the aquatic environment. In turn, in study [35], CWWs were dominated by *Proteobacteria* and *Bacteroidetes*. It has been indicated that their distribution depends on several factors, including the presence and concentration of petroleum hydrocarbons as well as metal contaminants and sulfate. Undoubtedly, the above-reported results show the microbiological risks related with wastewaters generated at car wash stations. It is important to note that biofouling development poses an additional challenge in the UF implementation and so far, it has not been investigated in studies focused on CWW treatment.

Given the fact that the fouling leads to increasing operational costs, it is highly recommended to take into consideration the effective operation of membrane cleaning. Numerous studies have argued that physical cleaning is used to remove reversible fouling while chemical cleaning is the most effective approach to removing an irreversible one [36,37]. Importantly, performing the literature review allowed us to point out that while a significant body of literature has focused on the application of membrane separation processes for CWW treatment, very little information is available on the cleaning of UF membranes. For instance, in [24], it has been demonstrated that the physical backflushing process is ineffective in cleaning ultrafiltration polyvinylidene fluoride (PVDF) and PES membranes fouled with CWW compounds. More precisely, it has been shown that physical backflushing restored the initial flow of PVDF and PES membranes at 30 and 50%, respectively. In turn, Kiran et al. [25] investigated the treatment of CWWs with the use of modified PES and cellulose acetate (CA) membranes. For cleaning the above-mentioned UF membranes, chemical cleaning with the use of sodium lauryl sulfate (SLS) solution has been performed. More precisely, the SLS solution flowed through membranes under a transmembrane pressure of 600 kPa for 45 min. Pinto et al. [23] applied physical and chemical cleaning to achieve the flux recovery of the cellulosic UF membrane. For this purpose, backwashing with water and cleaning with sodium hypochlorite (NaClO) aqueous solution have been performed. Finally, in [20], it has been found that using an alkaline detergent solution (pH = 11.5) recovers the maximum performance of PES membranes.

In recent years, touchless car washes have become popular. Usually, they are equipped with 2–3 car wash stations, and the wastewater produced is discharged through a clarifier and oil separator into the municipal sewage system. These car washes are manufactured in series; hence, the introduction and application of a separation system requires the acceptance of their owners (increased costs) and manufacturers. These car washes use an ion exchange system for softening the processed water, and part of it is also demineralized in the reverse osmosis (RO) process. The RO installation consists of a carbon filter and a spiral wound module (1–2 m^2^). A similarly simple construction is expected in the case of using the UF process for the CWW separation.

Undoubtedly, the implemented legal regulations require the recycling of part of the wash water. For this purpose, part of the CWWs could be filtered and used as rinsing water at the initial stage of car washing. Car washes operate irregularly; however, the sedimentation tank allows for averaging their composition and removing most of the contaminants. Car wash manufacturers also supply their own cleaning agents, which allows for obtaining a relatively constant wastewater composition. In a previous work [20], it was shown that in such a case, membrane fouling can be limited by performing cyclic membrane cleaning with alkaline agents used in car washes to remove insects. This allows for avoiding the use of agents that have not been approved for car washing. On the other hand, such agents can cause membrane damage. However, experimental investigations conducted for two years have clearly demonstrated that PES membranes are resistant to membrane cleaning with these agents [38].

It is essential to mention that at nights, car washes are usually not used, which allows for a periodic operation of the UF installation. However, it is not known whether such interruptions in the UF operation will promote the microorganisms’ development. In addition, car washes do not employ staff and, consequently, drivers wash their cars themselves. Due to the lack of continuous service control, it can be expected that for various reasons, there may be several-day interruptions in membrane cleaning. The results available in the literature are inconsistent. Hence, they do not clearly define the impact of the issues reported above on the service life of UF installation.

Consequently, in the context of research findings presented in the literature, the main aim of the current work was to investigate the treatment of the wastewater from a hand car wash with the use of two ultrafiltration PES membranes. The effectiveness of membrane cleaning performed daily and with breaks of several days was analyzed.

## 2. Materials and Methods

### 2.1. Feed, Ultrafiltration Unit and Operating Conditions

In the current study, the wastewater was collected from the settling tank of a hand car wash. Additionally, the fouling phenomenon was caused by the synthetic wastewater (pH = 7.6), which was a mixture of Turbo Active Green Foam (0.5%) and Hydrowax (0.2%) produced by EuroEcol (Łódź, Poland). These agents are used in car washes to create foam and for waxing the paint off of cars. Their composition is presented in study [22].

Two types (UE10–10 kDa and UE50–100 kDa) of commercial ultrafiltration PES membranes were used. The membranes were manufactured by TriSep Corporation (Goleta, CA, USA). Characteristics of the ultrafiltration unit and PES membranes have been presented in previously published works [20,22]. The UF process was carried out under a transmembrane pressure (TMP) of 0.3 MPa for 5–8 h per day. The feed flow rate was 1 m/s. Subsequently, the procedure of membrane cleaning was performed (Section 2.2). UF tests were conducted 5 days a week, while the installation was filled with distilled water 2 days a week. The membranes were tested for over two months.

### 2.2. Membrane Cleaning

In the proposed solution, CWWs are separated for several hours in the UF installation after overnight stabilization in the clarifier. Subsequently, the installation is cleaned with Insect solution (containing detergents and NaOH) and then rinsed with RO permeate, which remains in the module for at least several hours. Soaking the membrane in demineralized water leads to the osmotic rinsing effect, which reduces the intensity of the fouling phenomenon [39]. In the present study, to replicate these conditions, the procedure of membrane cleaning presented in Table 1 was performed. After CWW filtration was completed (5–8 h), the feed was removed by flushing 1 L of distilled water through the installation, and the module was rinsed with 0.5% Insect solution (pH = 11.5). The Insect concentrate supplied by the manufacturer (EuroEcol, Łódź, Poland) was diluted to this concentration in the washing station. Then, the Insect solution was removed and the installation was rinsed with distilled water, which was left in the installation overnight. After an overnight standstill, the installation was filled with a new portion of distilled water, and after determining the permeate flux for pure water, the CWW filtration was resumed. Finally, additional cleaning was carried out using P3 Ultrasil 11 solution (pH = 12) (SUTURAMED, Szczecin, Poland) and 0.5% H_3_PO_4_ acid solution (ChemLand, Stargard, Poland).

### 2.3. Analytical Methods

The membrane morphology and deposit composition were studied using an SU8020 (Hitachi High Technologies Co., Tokyo, Japan) scanning electron microscope (SEM). All samples were sputter-coated with chromium. The Hach cuvette tests (Hach Lange, Düsseldorf, Germany) were used to determine the composition of feed and permeate. Bacterial cultures were carried out on Petri dishes in appropriate media from BioMaxima (Lublin, Poland), as was presented in [40]. The analytical methods used for investigating the membrane characteristics and parameters of wastewaters used in the present study have been presented and discussed in detail in several previous works, e.g., [20,22].

## 3. Results and Discussion

The separation efficiency of the UE10 (10 kDa) and UE50 (100 kDa) membranes used for CWW treatment was evaluated in recent studies by our research group [20,22]. Hence, in the present work, the above-mentioned issue has been omitted.

### 3.1. Characteristics of Real Wastewater

Undoubtedly, the quality and content of CWWs are crucial parameters indicating possible treatment processes. Hence, in the present study, CWW characteristics have been determined. Overall, values of parameters such as pH and the concentration of phosphorus (TP) of tested wastewater are in agreement with those reported in the literature (Table A1). Indeed, the pH of CWWs was equal to 7.7, reflecting agents used for car washing [41], while TP was equal to 8.61 mg/L.

It can be noted that tested wastewater was characterized by the COD, equal to 845 mg/L. As discussed in [42], high values of COD are related to the presence of detergents that are used for the washing process.

In turn, turbidity values are related to the amount of particulate matter present in CWWs [41]. In the present study, turbidity of CWWs was equal to 37 NTU.

Bacterial cultures were carried out on the Petri dishes using non-selective media (R2A LAB-AGAR). The average number of bacteria was 2.4 × 10^5^ CFU/mL.

It is well accepted that the conductivity is directly related to the concentration of dissolved solids in CWWs. The CWWs tested in this study were characterized by the conductivity of 1172 µS/cm, while values of CWW conductivity reported in the literature were in the range from 28.1 ± 0.9 to 44130 ± 120 µS/cm. The results of this investigation show that the recorded value is very close to those noted in studies [15,33,43,44] (Table A1).

### 3.2. Separation of Synthetic Mixture

**Figure 2:**
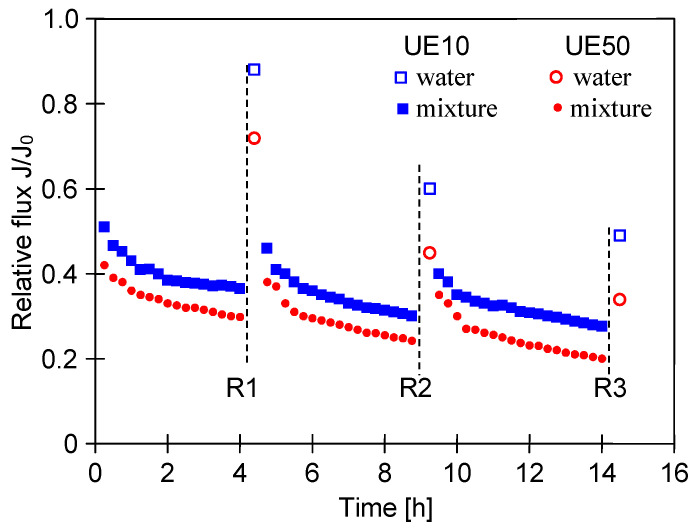
Changes in the relative permeate flux during separation of mixture (0.5% Turbo Active Green Foam + 0.2% Hydrowax). Maximum permeate flux: 360 L/m^2^h (UE10) and 900 L/m^2^h (UE50). R—membrane rinsing with distilled water.

In the car wash station, Turbo Active Green Foam was used to create foam and the cars were waxed using Hydrowax. The composition of the above-mentioned agents, containing nonionic and anionic surfactants, was presented in a previously published work [22]. Ultrafiltration experiments during a period of 14 h were conducted with the membranes UE10 and UE50 for the mixture of 0.5% Turbo Active Green Foam and 0.2% Hydrowax. As it was expected for both membranes, the maximum flux (360 L/m^2^h (UE10) and 900 L/m^2^h (UE50)) was decreased gradually with respect to time (Figure 2). It was observed during the experimental investigation that not only did the contaminants removed from cars cause the membrane fouling phenomenon, but also the agents used to wash the cars (such as waxes).

Although a severe fouling mechanism has been observed for both membranes, it should be noted that the relative flux of UE10 was higher than that noted for the UE50 membrane. It can be explained by the fact that membrane UE50 is characterized by the lager MWCO. This allowed the feed components to enter the membrane and block its pores. Fitting the experimental data from a previous work to Hermia’s model allowed us to determine that membranes with an MWCO of 100 kDa are more prone to intermediate blocking [22].

Since the fouling phenomenon determines the economic feasibility of a large-scale operation of membrane processes, the cleaning of membranes requires in-depth analysis [45]. From the findings of this study, it can be pointed out that cleaning with the use of distilled water was ineffective in retrieving the maximum water flux. Indeed, cleaning the UE10 membrane with the use of distilled water after 4, 9 and 14 h of the process run allowed us to achieve relative fluxes of 0.9, 0.6 and 0.5, respectively. With regard to the UE50 membrane, the noted relative fluxes were equal to about 0.72, 0.42 and 0.38. According to the results obtained from the above experiments, it can be concluded that in order to recover the maximum performance of the membranes, using chemical agents is required. As was demonstrated, the use of alkaline Insect solutions allowed us to remove these kinds of deposits from the membrane surface [20].

**Figure 3:**
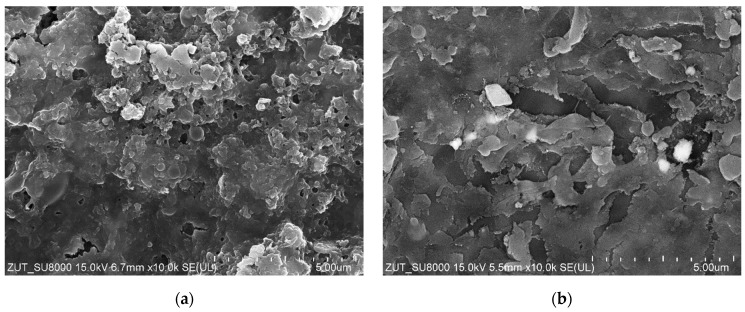
SEM images of the membrane surface after separation of Turbo Active Green Foam + Hydrowax mixture (Figure 1): (**a**) membrane UE10; (**b**) membrane UE50. After the UF process, membranes were rinsed only with water.

Results of the SEM analysis have clearly demonstrated that performing the UF process led to the formation of deposit on the membrane surface (Figure 3). The evidence above conclusively shows that it was the main reason for the flux decrease noted during the treatment process run.

### 3.3. Wastewater Separation

**Figure 4:**
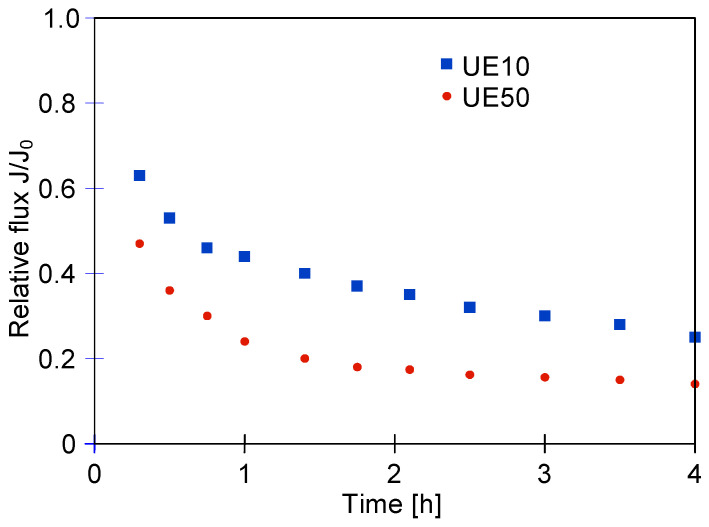
Changes in the relative permeate flux during treatment of real car wash wastewater.

Next, experiments were carried out to study the evolution of the UF process performance during the treatment of the real car wash wastewaters. Figure 4 demonstrates the changes in the relative flux for both membranes used. What can be clearly seen in this figure is the significant decrease in the process performance. Indeed, after 15 min, the relative flux was below 0.5. In turn, after 3 h, for the UE10 and UE50 membranes, it was equal to about 0.3 and 0.2, respectively. Worthy of note, the significant reduction in the performance of the UF process applied for the treatment of real CWWs was also reported in study [21]. The authors used membranes characterized by MWCO values from 1 to 50 kDa. For instance, it has been documented that for the UF with an MWCO equal to 50 kDa, the initial flux was 177.9 L/m^2^h, and it rapidly decreased to 68.9 ± 1.9 L/m^2^h in 10 min. In turn, after 1 h of the process run, the flux was equal to 35.2 ± 2.1 L/m^2^h. Furthermore, in [26], the separation of CWWs was investigated with the use of different commercial PES and polysulfone (PS) ultrafiltration membranes with MWCO values equal to 10, 25, 50 and 100 kDa. In the above-mentioned study, it was demonstrated that after 3 h of the process run, the relative flux was equal to or below 0.1. It should be pointed out that such results, without the development of a simple membrane cleaning method, exclude the use of UF systems for the separation of CWWs, especially in small car washes.

Since for the UE50 membrane (100 kDa), the relative flux was lower than that noted for the UE10 membrane (10 kDa), it can be expected that in the case of the UE50 membrane, the wastewater components entered through the pores, leading to intensive membrane fouling. Similarly, a greater decrease in the permeate flux for the UE50 membrane occurred during the separation of synthetic wastewater [22]; hence, the use of membranes with a lower MWCO value is more advantageous for the separation of CWWs.

**Figure 5:**
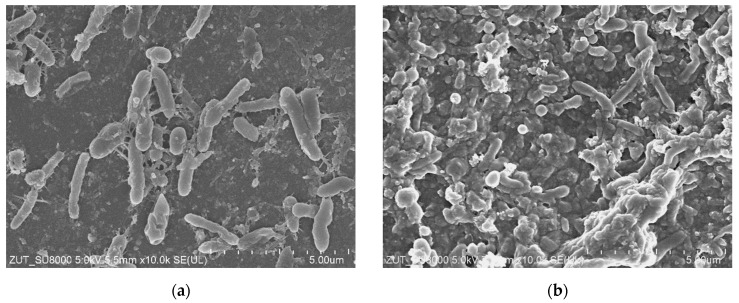
SEM images of the membrane surface after wastewater treatment: (**a**) membrane UE10; (**b**) membrane UE50.

SEM studies have demonstrated that the membrane’s surface after the treatment of real CWWs was covered with a significant amount of deposit (Figure 5). It is essential to mention that this finding is in agreement with that presented in study [22], wherein fitting the experimental data to Hermia’s model allowed us to show that during the separation of CWWs, with the use of UE10 and UE50 membranes, a cake layer is the key reason for the flux decrease.

Compared to synthetic wastewater (Figure 3), in the case of real wastewater, the deposits were formed not only by the suspension present in the CWWs, but also by bacteria. The high level of bacteria count (2.4 × 10^5^ CFU/mL) indicates that CWWs provide good conditions for microorganism development. As a result, an intensive development of biofouling may occur during UF installation. For this reason, in the next stage, UF tests were carried out over 2 months with repeated membrane cleaning.

**Figure 6:**
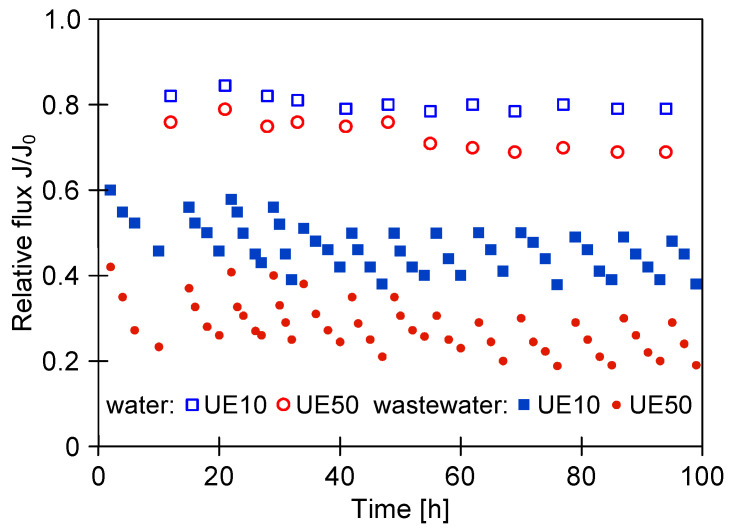
Changes in the relative permeate flux during long-term treatment of real car wash wastewater with cyclical washing using 0.5% Insect solution (pH = 11.5). Water—permeate flux after module washing (feed–distilled water).

Figure 6 shows the evolution of the relative permeate flux during the long-term (100 h) treatment of CWWs with the application of cyclical cleaning (every day) of UE10 and UE50 membranes. For this purpose, 0.5% Insect agent solution (pH = 11.5) has been used. It is immediately clear that the systematic cleaning of membranes with alkaline solution prevented a further decline in the process performance. The application of the above-mentioned agent provided a temporary increase in the flux at the levels of 0.5 and 0.3 for the UE10 and UE50 membranes, respectively.

**Figure 7:**
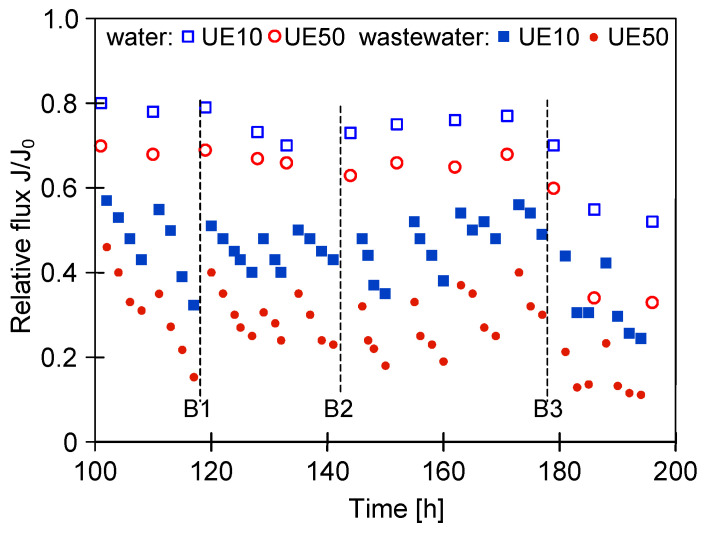
Effect of time break in the long-term UF process on the relative flux. B1—4-day break; B2—4-day break; B3—6-day break. Water—permeate flux after membrane washing with 0.5% Insect solution (30 min).

Another important issue that must always be considered is the impact of time breaks on the UF process performance. Hence, in the current study, the relative flux during the long-term process with breaks lasting a few days was investigated (Figure 7, B1–B3). Worthy of note, at the time, the installation was filled with distilled water. It can be seen that after the first break (B1), the maximum permeate flux for distilled water was approximately 0.8 and 0.7 for the UE10 and UE50 membranes, respectively. After the next breaks (B2 and B3), these values decreased significantly. During the last two series, despite the use of Insect solution, the relative flux values decreased to 0.53 (UE10) and 0.32 (UE50).

**Figure 8:**
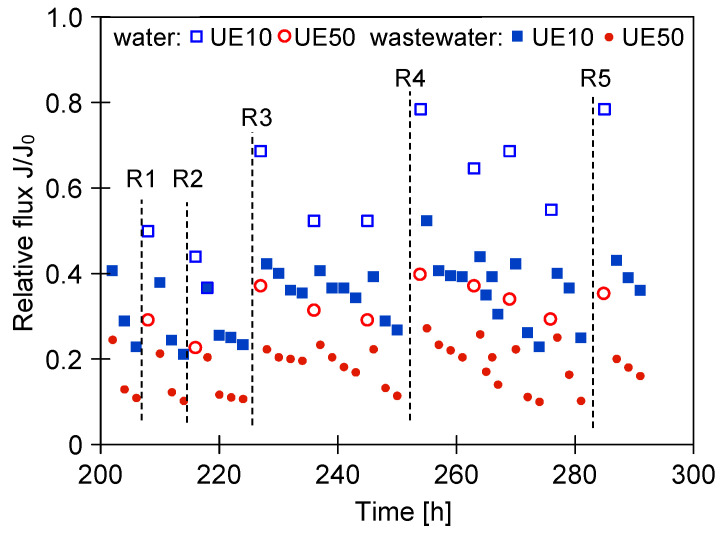
Results of intensified membrane cleaning. R1, R2—0.5% Insect 60 min. R3—0.5% P3 Ultrasil 11, 30 min. R4, R5—0.5% P3 Ultrasil 11, subsequently 0.5% phosphoric acid.

It has been found that as a consequence of breaks lasting a few days, the application of more intensive methods of membrane cleaning is required. Hence, many attempts have been made to restore the initial membrane performance (Figure 8). In the first stage, rinsing with Insect solution was extended to 60 min, which, however, did not lead to an increased flux (R1 and R2). Subsequently (R3), commercial membrane cleaner P3 Ultrasil 11 solution was used. This allowed us to clean the UE10 membranes; however, for the UE50 membranes, it was ineffective. For this reason, in the next steps, cleaning was carried out using P3 Ultrasil 11 and phosphoric acid (Table 1), which allowed us to increase the relative flux to a value of about 0.8 (UE10); however, the increase was only to the level of 0.35 for the UE50 membrane. This finding was probably due to the biofouling development which makes membrane cleaning more difficult. Hence, the results of this research support the indication pointed out in [46] that aqueous media favor microorganism adherence to the surface which is then coated by a layer of polymers. Undoubtedly, this phenomenon is the initial step leading to the biofilm formation. Indeed, it has been well documented that bacteria may colonize and form biofilms inside processing facilities [47,48,49].

**Figure 9:**
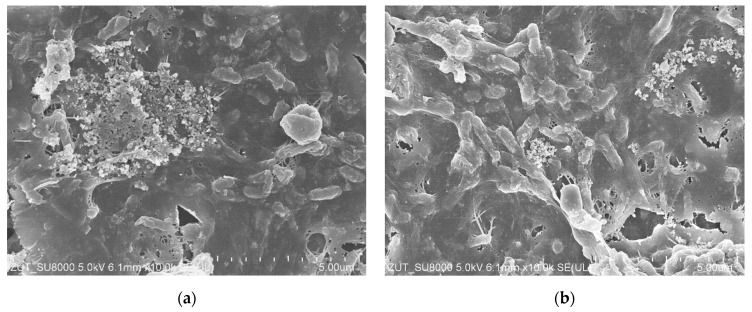
SEM images of the membrane surface after intensive membrane cleaning: (**a**) membrane UE10; (**b**) membrane UE50.

Interestingly, SEM studies performed after membrane cleaning confirmed that the membrane surface was covered with bacteria visible inside the deposit (Figure 9). Therefore, it is possible to hypothesize that the observed deposit was the main reason of the flux decline during the real CWW treatment. Importantly, Lau et al. [24] indicated that chemical cleaning may lead to undesirable damage to the membrane in long run. Nevertheless, the results obtained in the present study have demonstrated that the UE10 and UE50 membranes are resistant to the long-term exposure to bacteria as well as chemical agents. Indeed, the membrane damage has not been observed. However, it should be taken into account that the discussed touchless car washes operate without continuous service supervision. For this reason, the applied control systems for rinsing the installation cannot allow for operational breaks, such as those shown in Figure 7.

## 4. Conclusions

The present study was designed to evaluate the performance of the UF process used for the long-term treatment of real car wash wastewaters and the possibilities of effective membrane cleaning. This paper considers a system with a periodic operation of membranes dedicated to small touchless car washes. This paper has argued that since the permeate flux of PES membranes (10 and 100 kDa) significantly decreased during the process run, the chemical cleaning of membranes is required. For this purpose, an alkaline agent (Insect, pH = 11.5) used at car wash stations to remove insects from cars has been successfully applied. It has been found that systematic rinsing (every day) of membranes with the Insect agent prevented a further decrease in the process performance. However, with regard to the process run with breaks of a few days, it has been indicated that aqueous media favor microorganism adherence to the surface, which leads to the formation of biofilms inside processing installations. Therefore, performing more intense cleaning was required. For this purpose, Insect solution, P3 Ultrasil 11 agent and phosphoric acid have been used. One of the significant findings to emerge from this study is that the above-mentioned agents restored the relative flux to a value of 0.8 only for the membranes with an MWCO equal to 10 kDa. For this reason, eliminating the possibility of downtime is necessary. In addition, small touchless car washes are serviced periodically, which makes it difficult to use additional membrane cleaning, and the UF installation should be based on membrane cleaning with agents used for washing cars. Finally, the research has also indicated that the PES ultrafiltration membranes are resistant to the long-term exposure to bacteria and chemical agents. Undoubtedly, the results of this study have key implications towards applying the UF process for the treatment of car wash wastewaters.

## Figures and Tables

**Table 1:** The procedure of the UF membrane cleaning.

Step	Cleaning Agent/Volume	Time	Comment
flushing	distilled water/1 L	1 min	-
alkaline washing	Insect solution/1 L	30 min	
rinsing	distilled water/1 L	10 min	the installation filled with the portion of fresh distilled water
break	distilled water/1 L	15 h	-
			in the extended variant, washing was performed for 60 min
Additional cleaning			
alkaline washing	P3 Ultrasil 11/1 L	30 min	-
rinsing	distilled water/1 L	30 min	-
acid washing	phosphoric acid/1 L	30 min	-
rinsing	distilled water/1 L	30 min	-

## Data Availability

The data presented in this study are available on request from the corresponding author. The data are not publicly available due to the institutional repository being under construction.

## Appendix A

**Table A1:** Physical–chemical parameters of car wash wastewater reported in the literature.

pH [-]	SS [mg/L]	Turbidity [NTU]	COD [mg/L]	TP [mg/L]	Oil and Grease [mg/L]	Conductivity [µS/cm]	Ref.
8.5	4.2	1000	433	25.0	NI	NI	[2]
7.6	350	NI	460	NI	NI	604	[4]
6.5–8.0	NI	70–100	100–160	NI	NI	NI	[14]
6.3	114	NI	260	NI	<5.0 ppm	1070	[15]
7.3–7.6	3620	1590	4103	NI	1401	NI	[17]
7.3–7.6	5778	1920	4585	NI	2877	NI	[17]
8.0	5460	3770	8350	NI	4855	NI	[17]
7.3–7.6	3388	1500	2940	NI	1832	NI	[17]
7.9	NI	28.2	240	3.95	NI	NI	[20]
7.6	NI	19.1	181	4.61	NI	NI	[20]
7.3 ± 0.3	NI	NI	314.0 ± 9.4	NI	NI	729 ± 16	[21]
7.5 ± 0.2	260 ± 20	85 ± 8	85 ± 6	NI	<0.1	300 ± 10	[23]
7.1 ± 0.1	170 ± 10	44 ± 4	64 ± 6	NI	<0.1	370 ± 30	[23]
7.1 ± 0.1	100 ± 10	42 ± 4	59 ± 6	NI	<0.1	290 ± 20	[23]
6.51–8.74	NI	34.7–86.0	75–738	NI	NI	150.7–260.7	[24]
6.3–7.5	NI	132–140	150–175	NI	NI	1450–1570	[25]
NI	NI	186.6	700	NI	36	NI	[26]
6.13	80	149	419	NI	NI	497	[28]
6.58	800	89.9	80.5	NI	NI	318	[28]
6.42	NI	522	295	0.32	NI	404	[29]
4.66	NI	763	471.5	11.3	NI	509	[29]
6.2–8.7		128.7	155.6	NI	NI	NI	[30]
6.31	NI	58.5	387	12.2	NI	1232	[33]
7.45	NI	122	438	5.69	NI	2690	[33]
7.80	NI	60.4	871	2.59	NI	2495	[33]
8.4 ± 0.2	NI	388.3 ± 6.0	NI	NI	15.3 ± 3.1	124.7 ± 3.1	[35]
7.2 ± 0.2	NI	427.7 ± 35.7	NI	NI	49.7 ± 5.0	85.7 ± 2.5	[35]
7.4 ± 0.1	NI	137.3 ± 30.1	NI	NI	48 ± 5.6	30.0 ± 2.0	[35]
7.3 ± 0.1	NI	249 ± 3.6	NI	NI	25.7 ± 3.2	45.0 ± 4.1	[35]
7.3 ± 0.1	NI	253.3 ± 3.5	NI	NI	34.3 ± 1.5	49.7 ± 5.0	[35]
7.6 ± 0.1	NI	377.3 ± 5.0	NI	NI	36.7 ± 1.5	54.7 ± 2.5	[35]
7.4 ± 0.1	NI	234 ± 4.0	NI	NI	37.0 ± 1.0	60.3 ± 5.9	[35]
7.5 ± 0.2	NI	474.3 ± 17.5	NI	NI	30.3 ± 1.5	55.2 ± 4.3	[35]
7.5 ± 0.2	NI	263 ± 3.0	NI	NI	44.7 ± 1.5	51.7 ± 3.2	[35]
8.83 ± 0.22	1562.98 ± 3.01	88.19 ± 4.45	74.93 ± 2.70	2.58 ± 0.23	1394.81 ± 1.86	164.51 ± 3.59	[40]
8.98 ± 0.14	118.53 ± 0.54	61.19 ± 0.68	40.30 ± 0.08	2.18 ± 0.14	472.81 ± 1.31	153.60 ± 1.85	[40]
8.6 ± 0.2	3416.7 ± 1619.5	3649.2 ± 2149.7	1413.3 ± 327.6	7.0 ± 1.3	NI	376.1 ± 56.1	[41]
7.8 ± 0.4	1260.0 ± 910.7	1055.1 ± 731.8	990.0 ± 262.5	9.7 ± 2.6	NI	284.0 ± 53.4	[41]
7.6 ± 0.5	2929.2 ± 451.6	1912.5 ± 465.9	1337.3 ± 479.5	6.2 ± 3.3	NI	463.9 ± 89.6	[41]
6.96	NI	275.1	220	NI	NI	NI	[42]
8.0	2300	NI	560	NI	125	980	[43]
8.0	320	NI	500	NI	120	1000	[44]
7.08 ± 0.03	NI	170 ± 32.5	(480–1560) ± 207.3	NI	NI	7600 ± 2400	[50]
10.5	NI	312–420	7960–8190	NI	NI	4300	[51]
7.73 ± 0.85	85.0 ± 1.2	NI	190.0 ± 1.0	7.05 ± 2.20	68.0 ± 0.4	NI	[52]
7.43 ± 0.37	100.00 ± 0.62	NI	232.0 ± 0.6	9.40 ± 1.55	80.0 ± 0.3	NI	[52]
8.20 ± 1.66	325.0 ± 0.6.0	NI	485.0 ± 0.3	30.93 ± 0.31	85.0 ± 0.6	NI	[52]
7.4 ± 0.8	89 ± 54	103 ± 57	191 ± 22	NI	NI	469 ± 39.5	[53]
7.7 ± 0.6	502 ± 90.5	89 ± 16.5	241 ± 23.5	1.0 ± 0.5	6 ± 1	633 ± 125	[54]
8.2	55	28.1	82	NI	NI	NI	[55]
7.88 ± 0.04	NI	67.5 ± 1.8	506 ± 12	NI	NI	44,130 ± 120	[56]
NI	NI	185.80	305.15	17.30	NI	1130.18	[57]
7.7 ± 0.3	NI	1526 ± 348	42,255 ± 18,288	NI	127,301 ± 88,618	NI	[58]
7.2–7.6	NI	118–1400	610–2619	11.4–38.2	NI	419–2200	[59]
7.5	NI	NI	NI	NI	915	NI	[60]
6.90	1000	253	NI	NI	NI	NI	[61]
6.92–7.67	NI	362–450	NI	NI	NI	NI	[62]
7.2	112	NI	976	NI	88	NI	[63]
7.30 ± 0.2	NI	333 ± 10	357 ± 8	NI	NI	1430 ± 100	[64]
8.6 ± 0.1	1058 ± 19.8	382 ± 3.5	NI	NI	NI	122 ± 2.1	[65]
7.2 ± 0.2	16,262 ± 7.8	4000 ± 29.7	NI	NI	43	83.2 ± 1.9	[65]
7.5 ± 0.1	818 ± 3.5	372 ± 7.8	NI	NI	36	52 ± 2.8	[65]
7.7 ± 0.2	756 ± 2.1	455 ± 8.5	NI	NI	NI	50.6 ± 1.7	[65]
7.3 ± 0.2	612 ± 6.4	246 ± 7.8	NI	NI	NI	40.9 ± 0.3	[65]
7.5 ± 0.3	892 ± 13.4	109 ± 0.7	NI	NI	42	28.1 ± 0.9	[65]
7.9	940	NI	795.6	NI	30	NI	[66]
8.2	102	NI	323.7	NI	35	NI	[66]
7.87	116	NI	425	NI	94.6	NI	[66]
8.5	580	NI	504	NI	85	NI	[66]
7.9	110	NI	280	NI	40	NI	[66]
7.5	NI	NI	699	NI	539	NI	[67]
6.1 ± 0.4	NI	156 ± 45	626 ± 125	NI	NI	596 ± 155	[68]
7.31	NI	NI	625 ± 5	NI	NI	125	[69]
6.4	NI	NI	572	NI	NI	1600	[70,71]
6.08	NI	559	2640	NI	NI	1854	[72]
6.43	NI	733	4160	NI	NI	4090	[72]

NI—no information, SS—suspended solid, COD—chemical oxygen demand, TP—total phosphorus.
